# GRP78, a Novel Host Factor for SARS-CoV-2: The Emerging Roles in COVID-19 Related to Metabolic Risk Factors

**DOI:** 10.3390/biomedicines10081995

**Published:** 2022-08-17

**Authors:** Jihoon Shin, Shinichiro Toyoda, Atsunori Fukuhara, Iichiro Shimomura

**Affiliations:** 1 Department of Metabolic Medicine, Graduate School of Medicine, Osaka University, Suita, Osaka 565-0871, Japan; 2 Department of Diabetes Care Medicine, Graduate School of Medicine, Osaka University, Suita, Osaka 565-0871, Japan; 3 Department of Adipose Management, Graduate School of Medicine, Osaka University, Suita, Osaka 565-0871, Japan

**Keywords:** SARS-CoV-2 infection, COVID-19, GRP78, spike protein, ACE2, host factor, viral chaperone, viral co-receptor, cellular signaling/transcription, metabolic implication

## Abstract

The outbreak of coronavirus disease 19 (COVID-19), caused by the infection of severe acute respiratory syndrome coronavirus 2 (SARS-CoV-2), has resulted in an unprecedented amount of infection cases and deaths, leading to the global health crisis. Despite many research efforts, our understanding of COVID-19 remains elusive. Recent studies have suggested that cell surface glucose-regulated protein 78 (GRP78) acts as a host co-receptor for SARS-CoV-2 infection and is related to COVID-19 risks, such as older age, obesity, and diabetes. Given its significance in a wide range of biological processes, such as protein homeostasis and cellular signaling, GRP78 might also play an important role in various stages of the viral life cycle and pathology of SARS-CoV-2. In this perspective, we explore the emerging and potential roles of GRP78 in SARS-CoV-2 infection. Additionally, we discuss the association with COVID-19 risks and symptoms. We hope this review article will be helpful to understand COVID-19 pathology and promote attention and study of GRP78 from many clinical and basic research fields.

## 1. Introduction

Severe acute respiratory syndrome coronavirus 2 (SARS-CoV-2) is responsible for the pandemic of coronavirus disease 2019 (COVID-19), which has caused over 536 M cases and more than 6.3 M deaths worldwide, leading to a global health crisis [1,2]. SARS-CoV-2 can be detected from various specimens, such as nasal/pharyngeal swabs, saliva, feces, and blood [3,4]. COVID-19 symptoms vary between patients roughly graded as asymptomatic, mild, moderate, severe, and critical. The patient infected by SARS-CoV-2 shows various signs including fever, cough, breath shortness, fatigue, muscle pain, diarrhea, dizziness, and loss of smell and taste. In the severe cases, the incidence of pneumonia, acute respiratory distress syndrome (ARDS), sepsis, and multiple organ failure critically contributes to serious progression and mortality of COVID-19 [1,2]. Cumulative data have shown that patients with older age, obesity, diabetes, visceral adiposity, and some types of cancers are more vulnerable to severe COVID-19 [5,6,7,8,9]. Despite the many advances made in understanding the pathology of COVID-19, little is known about the molecular basis and therapeutic targets.

**Figure 1:**
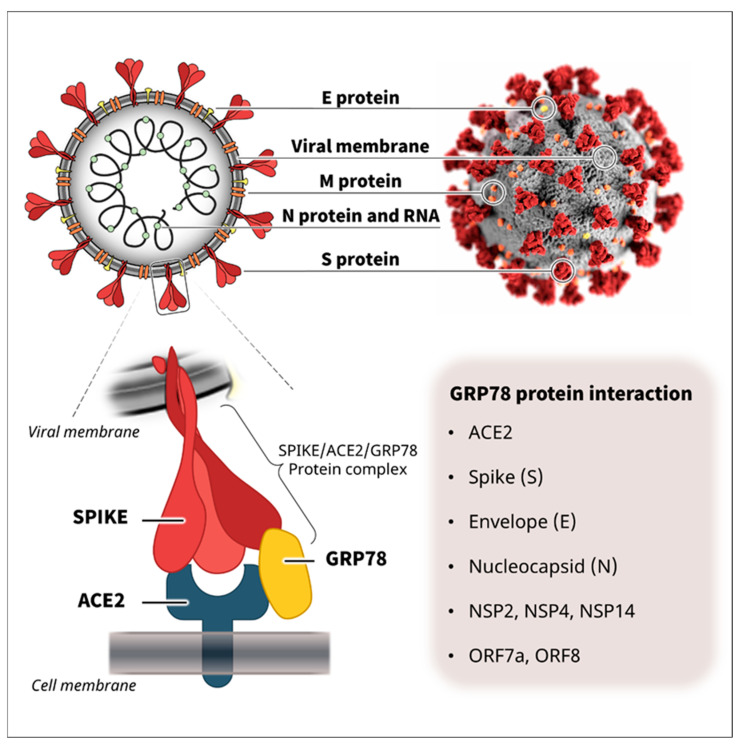
SARS-CoV-2 structure and protein interactions. SARS-CoV-2 is enveloped by a lipid bilayer membrane and consists of several viral proteins [24,25,26]. Recently, we and another research group independently reported that GRP78 functions as a host co-receptor for SARS-CoV-2 infection via complex formation with SARS-CoV-2 spike protein and host receptor ACE2 [12,15]. Cumulative protein interactome data have shown that GRP78 can also interact with other viral proteins of SARS-CoV-2, such as the spike (S), envelope (E), nucleocapsid (N), NSP2, NSP4, NSP14, ORF7a, and ORF8 [16], suggesting its possible involvement in other lifecycle stages and pathology of SARS-CoV-2. The upper right illustration of SARS-CoV-2 was created at the Centers for Disease Control and Prevention (CDC).

Viruses such as SARS-CoV-2 cannot survive without the support of a host environment. SARS-CoV-2 utilizes angiotensin-converting enzyme 2 (ACE2) as the major host receptor during infection [10,11], but the low expression and limited cell distribution suggested the possible involvement of other host factors [12]. Glucose-regulated protein 78 (GRP78) is an important molecular chaperone that regulates protein homeostasis in the endoplasmic reticulum (ER). However, emerging studies have indicated that GRP78 is also identified on the cell surface and involved in various biological processes beyond the function in the endoplasmic reticulum, such as cell signaling, inflammation, apoptosis, and viral infection [13,14]. Recently, we and another research group independently reported that glucose-regulated protein 78 (GRP78) functions as a host co-receptor for SARS-CoV-2 infection [12,15], and cumulative protein interactome data have shown that GRP78 can also interact with other viral proteins of SARS-CoV-2 (Figure 1) [16]. Given the physiological and pathological functions of GRP78 in the regulation of biological processes, such as protein homeostasis, cellular signaling, and transcription activity [13,14,17,18,19,20], it could possibly contribute to several stages of the viral life cycle and pathology of SARS-CoV-2. Furthermore, the expression of GRP78 is significantly associated with the risk factors for severe COVID-19 symptoms, such as older age, obesity, diabetes, and lung cancer [12,21,22,23]. Therefore, the understanding of GRP78 roles from diverse perspectives will provide a better comprehension of COVID-19 pathology and offer a novel viewpoint for the future therapeutic target.

## 2. Cell Surface GRP78 as a Co-Receptor for SARS-CoV-2 Infection

**Figure 2:**
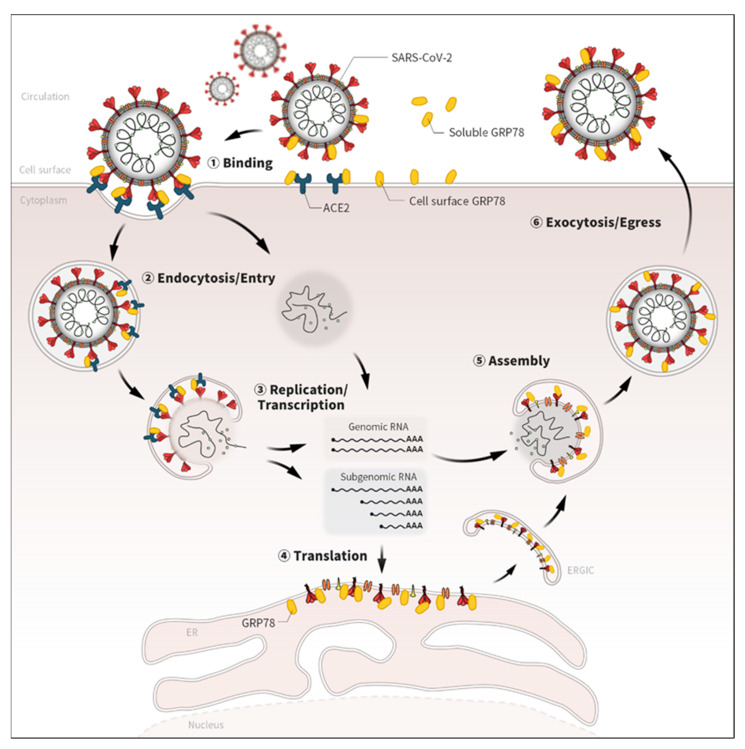
Potential roles of GRP78 in the life cycle of SARS-CoV-2. Emerging evidence has shown that GRP78 directly binds with several viral proteins of SARS-CoV-2 [12,15,16,18]. Given the localizations and biological roles, GRP78 could also contribute to various life-cycle stages of SARS-CoV-2, not only the binding but also the endocytosis/entry, replication, translation, assembly, and exocytosis/egress processes.

GRP78, also referred to as BiP/HSPA5, is well known as a molecular chaperone conventionally residing in the endoplasmic reticulum (ER) and plays an important role in regulating protein homeostasis, such as protein folding, assembly, stability, and degradation [13,14]. However, under stress conditions, GRP78 is overexpressed and detected on the cell surface, where it serves as a binding partner for various ligands and contributes to the pathology of many human diseases, such as infections and cancers [13,14]. The localization of GRP78 protein is attributed to its intrinsic and extrinsic properties [27,28,29]. GRP78 contains an N-terminal signal peptide, which targets it into the ER during the process of protein translation [27]. The C-terminal KDEL (Lys-Asp-Glu-Leu) sequence of GRP78 protein is recognized by the KDEL receptor, which controls the retrograde transport of chaperone proteins from the Golgi to the ER apparatus [28]. The overexpression of GRP78 protein in response to ER stress disrupts its retention in the ER, which is associated with the saturation and dispersion of KDEL receptors, causing the escape of GRP78 form the ER and relocation to the cell surface [30,31,32]. The cell surface GRP78 (csGRP78) has binding affinity not only for many endogenous molecules but also those of exogenous origin, including various viral proteins [13,14]. Two recently published independent studies have shown that csGRP78 can act as a co-receptor for the SARS-CoV-2 spike protein with the receptor ACE2 [12,15]; csGRP78 alone did not promote the binding of SARS-CoV-2 spike protein on the cell surface, but in combination with ACE2 expression it could enhance the binding and accumulation of SARS-CoV-2 spike protein to the cells [12]; the amount of SARS-CoV-2 spike protein on the cell surface was dependent on the expression level of GRP78 [12], suggesting the significant role of csGRP78 as a co-receptor with ACE2 for SARS-CoV-2 infection. Mechanistically, csGRP78 directly interacts with SARS-CoV-2 spike protein and forms a protein complex with the host cell receptor ACE2 on the cell surface, which facilitates the entry into the target cells [12,15]; the host factor efficacy of csGRP78 for SARS-CoV-2 infection was associated with the stabilization of ACE2 protein expression on the cell surface [15]. The role of csGRP78 as a host co-receptor has been reported in other coronaviruses such as MERS-CoV and bCoV-HKU9; the binding with the spike proteins promotes the attachment and entry of the viruses into the host cells [20]. Similarly, other virus strains, such as dengue virus, coxsackievirus A9 (CAV-9), Japanese encephalitis virus (JEV), and Tembusu virus (TMUV), likely utilize csGRP78 as the host cell receptor or binding partner, facilitating the infection of target cells [33,34,35,36,37]. Hence, cell surface-expressed/localized GRP78 may play an important role in SARS-CoV-2 infection as a co-receptor or binding partner through direct interaction with the spike protein and viral receptor ACE2 (Figure 2). Physiologically, GRP78 as a molecular chaperone controls the protein homeostasis of secretory and membrane proteins, such as ACE2, and is important for their proper folding, assembly, maturation, and stability [12,13,14,15]. GRP78 is also essential for regulation of the unfolded protein response (UPR) under pathological stress conditions; it actively interacts with misfolded proteins presumed to be harmful for the cells [13,14]. GRP78′s binding affinities—physiologically for endogenous ACE2 and pathologically for exogenous viral proteins—likely intensify the entry of SARS-CoV-2 on the cell surface of target cells.

## 3. GRP78 as a Viral Chaperone for SARS-CoV-2

SARS-CoV-2 is a strain of coronavirus that is enveloped by a lipid bilayer membrane and consists of several viral proteins [24,25,26]: four structural proteins—the spike (S), envelope (E), membrane (M), and nucleocapsid (N) proteins; 16 non-structural proteins (NSP1–16), cleavage products of ORF1a and ORF1ab; and 11 accessory proteins: ORF3a, ORF3b, ORF3c, ORF3d, ORF6, ORF7a, ORF7b, ORF8, ORF9b, ORF9c and ORF10. These viral proteins are important for maintaining the viral life cycle and infectivity of SARS-CoV-2, but the synthesis and replication mechanisms are still largely unknown. Viruses cannot replicate on their own; thus, they need to exploit the host cell’s machinery to reproduce the viral proteins [11,20,24,38,39]. Endoplasmic reticulum (ER) is the central place for the production, folding and assembly of membrane and secretory proteins [13,14,15]. GRP78 is an ER-located molecular chaperone and plays critical roles in protein folding, assembly, and homeostasis not just for endogenous proteins but also for those of exogenous origin, such as viral proteins [13,14,15,17]. Viruses, including dengue virus, Japanese encephalitis virus, human cytomegalovirus, Ebola virus, and hepatitis B virus, abduct GRP78 and exploit its chaperone function for the proper synthesis of their viral proteins [35,40,41,42,43,44]. This viral trait also likely applies to the case of the SARS-CoV-2 virus; overexpressed spike protein of SARS-CoV-2 directly binds with the endogenous GRP78 in mammalian cells [12,15]. The depletion of GRP78 expression and activity by AR12, a potent inhibitor of PDK1/AKT signaling and ATPase activity, decreased the production of SARS-CoV-2 spike protein [19]. Since the outbreak of COVID-19 in December 2019, there have been many efforts to improve our understanding of the protein properties and interactions of SARS-CoV-2. The cumulative data on SARS-CoV-2 protein interactomes have indicated that GRP78 may physiologically interact with several viral proteins of SARS-CoV-2, such as the spike (S), envelope (E), nucleocapsid (N), NSP2, NSP4, NSP14, ORF7a, and ORF8 [16]; the S, E, and N proteins that are commonly found in all coronavirus strains, and essential components of the viral structure and infectivity. Thus, these viral proteins are usually targeted by anti-viral therapeutic approaches [25,26]. NSP2, NSP4, and NSP14 along with other factors form a replication–transcription complex (RTC) and are involved in genome replication and early transcription regulation [26]. ORF7a suppresses the type I interferon response, increases the immune and cytokine responses, and contributes to the replication of SARS-CoV-2 [45,46,47]. ORF8 is involved in the immune evasion and inflammatory response of SARS-CoV-2 [48,49,50]. Collectively, GRP78 might serve as a host viral chaperone for SARS-CoV-2 proteins, such as S, E, N, NSPs, and ORFs, and facilitate the folding, assembly, and maturation of the viral proteins contributing to the pathology of COVID-19. GRP78 can bind with a wide range of endogenous and exogenous proteins accumulated in the ER—as a key regulator of unfolded protein response (UPR)—especially under stress conditions [14]. The broad spectrum of binding affinity for proteins likely works as an advantage for viruses; thus, they might have evolved to utilize GRP78 as their viral chaperone, facilitating viral replication, assembly, and egress (Figure 2).

## 4. GRP78 as a Mediator of Cellular Signaling for SARS-CoV-2

**Figure 3:**
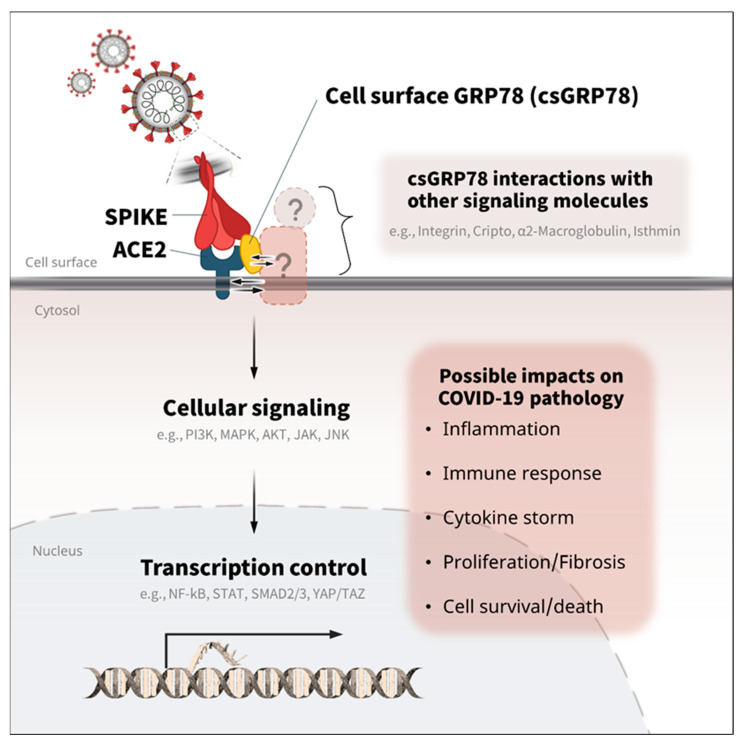
Potential roles of GRP78 in the cellular signaling and transcription of COVID-19. GRP78 is associated with various cellular signaling and transcription activities involved in inflammation, cytokine release, fibrosis, and cell survival/death [13,51,52,53,54,55]. Thus, the direct and/or indirect activation of GRP78 with ACE2 and other signaling molecules (integrin, Cripto, α2-Macroglobulin, isthmin, etc.) might be associated with the pathology of COVID-19 via the linked intracellular signaling (PI3K, MAPK, ATK, JAK, JNK, etc.) and transcription control (NF-kB, STAT, SMAD2/3, YAP/TAZ, etc.) [51,53,56,58,59,60].

GRP78 has long been considered as a chaperone protein responsible for protein homeostasis and stress regulation in ER, but accumulated studies have shown that it can also serve as a signaling mediator on the cell surface and is involved in many pathological processes such as cellular inflammation, apoptosis, survival, and proliferation [13,51,52,53,54,55]. The interaction of csGRP78 with other membrane proteins and ligands transduces various cellular signaling and transcriptional activity. The interaction of csGRP78 with α2-Macroglobulin (α2M*) activates MAPK- and AKT-dependent signaling and the NF-κB pathway, promoting cellular proliferation with reduced apoptotic response [51,56]; the binding also regulates PDK1 signaling and c-MYC activity associated with cell proliferation [57]. csGRP78 also controls transcriptional coactivators YAP and TAZ—integral components of the Hippo signaling pathway that controls cellular mechanical and cytoskeletal cues—to regulate the mobility and invasiveness of tumor cells [54]. The complex formation of GRP78 with Cripto on the cell surface is important for the downstream cellular events, such as MAPK/PI3K signaling and the SMAD2/3 pathway, decreasing cell adhesion and proliferation [53]. The interaction activates the JAK/STAT pathway in association with cell survival and proliferative properties [58]. These csGRP78-associated cellular signaling and transcriptional pathways play an important role in the development of various pathologies. NF-κB and JAK/STAT3 are well known for their proinflammatory and apoptotic responses [13,51,52]. SMAD2/3 is well established for its role in fibrosis formation [13,53]. The PI3K/AKT/MAPK and YAP/TAZ pathways are crucial for cell survival and proliferative processes [13,53,54,55]. Of note, the SARS-CoV binding to ACE2 receptor induces inflammatory responses and cytokine release in part via the RAS/RAF/MAPK pathway [59]. In addition, the SARS-CoV-2 spike protein activates cell surface signaling molecules—integrins—via the direct binding, which is involved in the infection of SARS-CoV-2 [60]. Therefore, the activation of cell surface GRP78 via the direct binding with SARS-CoV-2 or the indirect stimuli linked with other signaling molecules, such as integrins, might change these signaling and transcriptional pathways affecting COVID-19 pathology (Figure 3). Further experimental assessments are needed to understand the signaling and transcriptional impacts of csGRP78 in SARS-CoV-2 infection and its contribution to the development of COVID-19 pathogenesis, such as the inflammation, cytokine storm, cell death and fibrosis responses.

## 5. Soluble GRP78 as a Possible Binding Partner for SARS-CoV-2 in Circulation

GRP78 is found not only in cellular regions—the ER compartment and cell surface—but also in circulation as a soluble form of GRP78 [21,22,61,62]. The soluble GRP78 level increases with SARS-CoV-2 infection, which is more evident in patients with pneumonia [62]. The circulating level of GRP78 is significantly associated with metabolic disorders, such as obesity and diabetes [61]. The expression level of circulating GRP78 is also elevated in patients with lung cancers [21,23]. These pathological and disease conditions are well known to increase the risk of COVID-19 severity and mortality [63]. Our recent study showed that the treatment of soluble GRP78 with ACE2-expressing human lung epithelial cells, a mimetic of the circulating GRP78 in vitro, facilitated the cellular binding and accumulation of SARS-CoV-2 spike protein in the target cells [12], suggesting the possible impact of soluble GRP78 on SARS-CoV-2 infection. It has been suggested that GRP78 enhances the stability and expression of the binding proteins; cell surface GRP78 can stabilize ACE2 or ADMA17 protein on the cell surface and is associated with virus infectivity [15,64]. GRP78 was also reported to promote the entry of binding substances into the cells via the endocytosis pathway [65,66]; the binding of isthmin with GRP78 on the cell surface facilitates the internalization of the complex through clathrin-dependent endocytosis and triggers cellular apoptosis [65]; the interaction of dentin matrix protein 1 (DMP1) with GRP78 at the plasma membrane induces endocytic trafficking, which contributes to the osteogenic differentiation of human stem cells [66]. β-coronaviruses, such as SARS-CoV-2, are co-localized and-released with GRP78 during infection by the virus [18], suggesting potential roles in the exocytosis/egress of SARS-CoV-2. Collectively, it can be speculated that soluble GRP78 may surround SARS-CoV-2 via direct binding with the spike protein in circulation. The complex formation of soluble GRP78 with viral particles in circulation or at plasma membrane might enhance the stability of the virus and facilitate its attachment to and endocytosis by the target cells (Figure 2. These properties could also lead to SARS-CoV-2 tropism and multi-organ infection beyond the respiratory tract, such as the kidneys, heart, liver, adipose tissues, brain, and pancreas [67,68,69,70]; the enhancement of stability and tropism might also contribute to prolonged symptoms after SARS-CoV-2 infection and recovery, termed long COVID [71,72,73]. Further experimental and clinical studies need to be performed to verify these points.

## 6. Other Pathological Traits of GRP78 for SARS-CoV-2

There are many other pathological aspects of GRP78 associated with the SARS-CoV-2 life cycle and development of COVID-19 symptoms. GRP78 is induced when the ER is under stress conditions such as the excessive overload of protein synthesis [13,14]. The induction of GRP78 in association with UPR response under stress can inhibit cell apoptosis and promotes pro-survival processes in the cells [13,14,17]. Overexpression of GRP78 is also observed in many viral infection cases [17,40,44], which might support the long-lasting residence of SARS-CoV-2 with massive production of viral proteins in the host. Of note, the overexpression of GRP78 is also found in the case of SARS-CoV-2 infection, which might be associated with ER stress by excessive production of the viral proteins; high expression of GRP78 was detected in pneumocytes, macrophages, and the circulation [62,74]. The expression of GRP78 is highly relevant to apoptotic, immune, and fibrotic responses in the lung [75,76]; Grp78 heterozygosity (Grp78+/− mice were strongly protected from bleomycin-induced fibrosis and exhibited better lung function [75]; inactivation of GRP78 attenuated endothelial inflammation and permeability against acute lung injury [76], suggesting its possible involvement in lung pathology and function of COVID-19 patients. GRP78 can stabilize ADAM17 on the cell surface, which was reported to enhance the previous strain of coronavirus, SARS-CoV, by promoting the shedding of the viral receptor ACE2 [64]. GRP78 also likely plays a role in the cell surface expression of ACE2 protein, presumably via direct binding and stabilization [15]. After the infection, GRP78 is released together with the replicated SARS-CoV-2 viruses from the host cells via the lysosomal exocytic pathway, suggesting its involvement in the replication, egress and stabilization of the virus [18]. GRP78 can interact with the major histocompatibility class one (MHC-I) molecule on the cell surface [77], which might contribute to the ability of antigen presentation and subsequent immune response in SARS-CoV-2 infection. Serious co-infections and secondary infections by several bacteria and fungi have been reported in COVID-19 patients, which contribute to the severity of COVID-19 as well as poorer outcomes [78,79,80]. GRP78 acts as a host receptor for fungal pathogen *Rhizopus oryzae*, the most common etiologic species of Mucorales, and mediates the invasion and damage of human endothelial cells [81]; GRP78 binds with Pneumocystis carinii on the cell surface of lung epithelial cells, mediating the attachment [82]; these studies suggest that GRP78 might be involved in cases of co-infection and secondary infection in COVID-19 patients. GRP78 is well conserved and presented from bacteria (Dna K: bacterial GRP78 homology) to humans (GRP78/BiP/HSPA5) [83]. The bacterial GRP78 is essentially required for proper bacterial growth and mRNA/protein [84], and is a therapeutic target for both bacterial and viral infection [85]. Of note, SARS-CoV-2 is detected alive in feces specimens from COVID-19 patients [3,4]; SARS-CoV-2 has bacteriophage-like behavior, replicating in bacteria [86]; SARS-CoV-2 infection impairs gastrointestinal (GI) microbiota, which is associated with COVID-19 severity [87]. These data suggest that the interaction of GRP78 with SARS-CoV-2 could contribute to replication/transmission by GI/fecal bacteria, impair the microbiota, and affect the severity of COVID-19. Altogether, these biological features of GRP78 potentially support the long-lasting life cycle and pathological development of SARS-CoV-2 in the host. The pathological roles of GRP78, such as pro-survival and inflammatory and/or apoptotic responses [13,14,17,32,65,88], may be dependent on the binding partner/properties or the duration, progress, and levels of SARS-CoV-2 infection.

## 7. The Association of GRP78 with COVID-19 Risks

**Figure 4:**
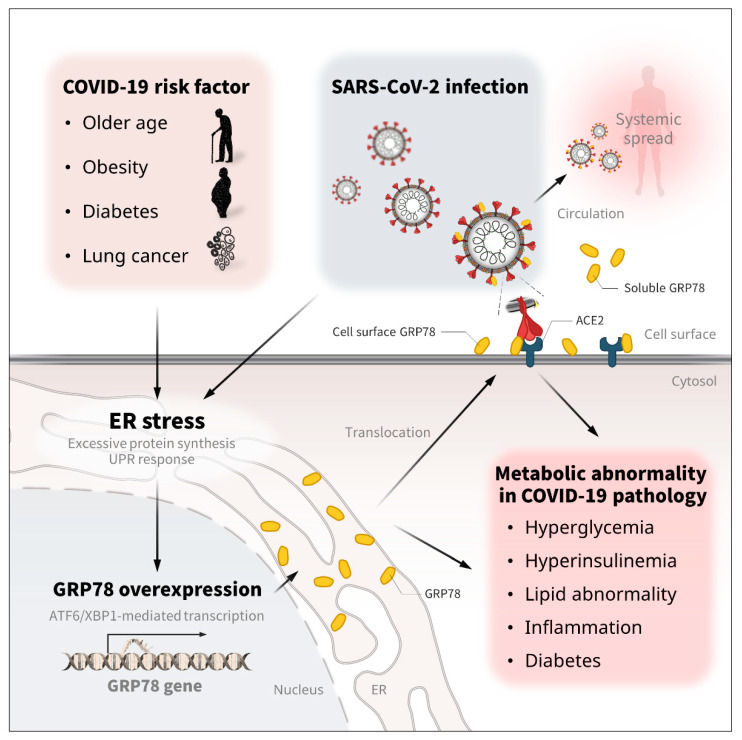
The regulation and metabolic association/implication of GRP78 in COVID-19. Older age, obesity, diabetes, and some types of lung cancer are known to be significant risk factors for COVID-19 severity and mortality [5,6,7,8,9]. Theses pathological conditions increase the expression of GRP78 [21,22,23,61]. SARS-CoV-2 infection is also associated with the overexpression of GRP78 in the lung and circulation [62,74]. The excessive protein production and UPR response in the ER compartment is attributed to GRP78 overexpression via ATF6/XBP1 transcription activity [12,89,90], which triggers the translocation of GRP78 to the cell surface and the circulation [30,31,32]. The excessive expression of GRP78 contributes to metabolic diseases, such as hyperglycemia, hyperinsulinemia, liver steatosis, abnormal lipid control, inflammation, insulin resistance, and diabetes [91,92,93]. Therefore, the infection- and stress-induced GRP78 expression might in part account for the metabolic abnormalities of COVID-19.

SARS-CoV-2 infection does not cause severe and serious symptoms in all patients, but patients with older age, obesity, diabetes, lung cancer, and some other metabolic abnormalities are more vulnerable [5,6,7,8,9]. Recently, we showed that the expression of GRP78 is abundant in adipose tissues, especially in the visceral regions, and augmented by the conditions of older age, obesity, and diabetes; the overexpression of GRP78 in adipocytes was attributed to hyperinsulinemia, which is usually found in older age and obese and diabetic conditions, and is significantly associated with the stress-responsive transcription factor XBP-1s; glucose levels had little impact on the regulation of GRP78 in adipocytes, suggesting that insulin resistance is a major cause of GRP78 overexpression in adipose tissue [12]. ATF6 activation is also associated with the elevated expression of Grp78 [89,90]. The circulating level of GRP78 is also significantly correlated with obesity, diabetes, and other metabolic syndromes [22,61]. GRP78 is also well known for its high expression in many types of cancers, especially in lung cancers; the circulating levels markedly increase in association with the progression and severity [21,23]. These pathophysiological human health and disease conditions—older age, obesity, diabetes, some types of cancer—likely create environments for the induction and activation of GRP78 in intracellular, extracellular, and circulation areas, which potentially makes patients more vulnerable to COVID-19 (Figure 4).

## 8. Metabolic Implications of GRP78 in COVID-19

In recent years, it has become apparent that SARS-CoV-2 infection can have harmful effects far beyond the lungs; COVID-19 is well known for causing respiratory disease but can also trigger metabolic abnormalities [63,70]. However, the molecular mechanisms remain largely elusive. SARS-CoV-2 infection is associated with the elevated expression of GRP78 in the lung and circulation [62,74], and presumably other infected organs and cells such as liver, adipose tissue and immune cells might exhibit similar overexpression of GRP78 through the virus-induced ER stress and unfolded protein response. The pathological implication of GRP78 overexpression in metabolic diseases has been well established in many previous studies; the expression of GRP78 is significantly increased in the adipose tissue of patients with obesity and diabetes [12]; the circulating level of GRP78 is a molecular marker of several metabolic diseases, including obesity, diabetes, and atherosclerosis [22,61]; the heterozygosity of the Grp78 gene increases energy expenditure and is protective against high-fat diet (HFD)-induced hyperinsulinemia, hyperglycemia, liver steatosis, and adipose inflammation with adaptive UPR [89]. Macrophage-selective ablation of the Grp78 gene improves insulin sensitivity and glucose metabolism in muscle and reduces inflammation in adipose tissue [91]; covalent inhibition of GRP78 by celastrol inhibits lipid accumulation in liver and adipose tissue with reduced ER stress and inflammation [92]. Overexpression of Vaspin, an endogenous antagonist of cell surface GRP78/MTJ-1 complex, ameliorates diet-induced obesity, glucose intolerance, and/or hepatic steatosis with improved ER stress [93]. These research findings have suggested that infection- and stress-induced GRP78 expression might contribute to the metabolic abnormalities of COVID-19 (Figure 4); thus, the detailed relationships should be investigated in future studies.

## 9. Therapeutic Strategies Targeting GRP78

Given the importance of GRP78 in various biological processes, the interaction with SARS-CoV-2 protein, and the association with COVID-19 pathological risks, targeting either the activity or the expression of GRP78 could be an efficient therapeutic/preventative strategy to dampen the multiple stages of the SARS-CoV-2 life cycle including the binding, entry, replication, egress, and stability. In fact, blocking or depleting cell surface GRP78 by specific antibodies reduced the infectivity of related viruses including SARS-CoV-2 [15,17,20,34]. The ablation of GRP78 expression by siRNA decreased not only the entry of several viruses but also the production of viral proteins in the host cells [17,20,37]. Other inhibitory molecules related to the expression and/or activity of GRP78, such as subtilase cytotoxin (SubAB), AR12 and epigallocatechin gallate (EGCG), similarly abrogated the entry and/or replication process of viruses in the host cells [17,19,35,42]. Reducing metabolic stress by pharmacological and lifestyle interventions effectively lowers the expression of GRP78 and may be helpful for improving COVID-19 symptom; Anti-diabetic drugs, such as metformin and SGLT2 inhibitor, or lifestyle changes, such as calorie restriction and exercise, reduced the expressions of GRP78 in adipose tissues [12]; appropriate control of metabolic abnormalities by diabetic drugs may improve the severe outcomes of COVID-19 [12,94]. There are also in silico/virtual analyses of GRP78 protein that suggest some putative inhibitory peptides and molecules [95,96] and potential interaction changes between GRP78 and SARS-CoV-2 strains, which might be helpful for screening the therapeutic targets of COVID-19 [97]. Targeting GRP78 under stress conditions could be a promising therapeutic approach to preventing or inhibiting the infection/lifecycle of SARS-CoV-2 and the development of COVD-19 pathology.

## 10. Conclusions

**Figure 5:**
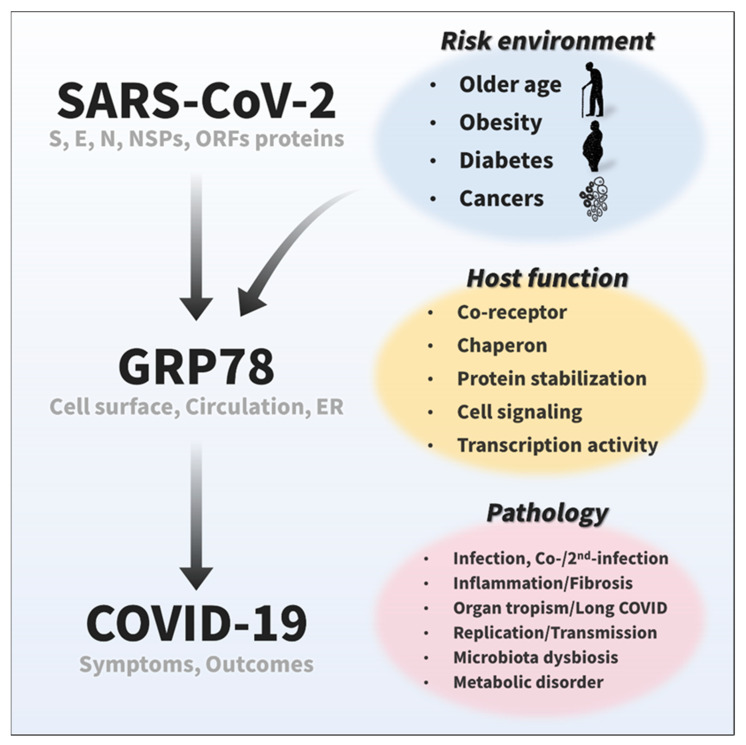
Emerging and potential roles of GRP78 in COVID-19. Recent studies have suggested that GRP78 on the cell surface and in circulation acts as a host co-receptor for SARS-CoV-2 infection [12,15], and cumulative protein interactome data have shown that GRP78 also could interact with other viral proteins of SARS-CoV-2 [16]. The expression of GRP78 is highly relevant to COVID-19 risk factors, such as older age, obesity, diabetes, and cancer [12,21,22,23,61]. Given its significant roles in a wide range of biological processes, including protein homeostasis and cellular signaling, GRP78 possibly plays an important role in various viral life cycle stages (binding, entry/endocytosis, replication, translation, assembly, egress/exocytosis, stabilization, etc.) and pathology (inflammation, fibrosis, organ tropism, long COVID, co-/2nd-infection, microbiota, metabolic disorders, etc.) of SARS-CoV-2.

In conclusion, we suggest that GRP78 may be a multi-functional host factor involved in various steps of the SARS-CoV-2 life cycle as co-receptor, chaperone, protein stabilizer, and mediator of cellular signaling and transcription (Figure 5). These emerging and potential roles of GRP78 possibly contribute to the pathology of COVID-19, such as not only the infection, but also inflammation, fibrosis, egress, organ tropism, long COVID, co-/2nd-infection, and metabolic abnormalities. Its expression is highly relevant to the risk factors for severe symptoms and outcomes in COVID-19 patients, such as aging, obesity, diabetes, and lung cancer. Thus, targeting GRP78 could be an efficient preventative and therapeutic strategy for COVID-19 pathology. We hope that this review promotes further attention to and study of GRP78 from many clinical and basic science fields. Sophisticated and detailed research should be performed to assess the potential roles of GRP78 and its associations with COVID-19 pathology, risk, and metabolic impact.

## Data Availability

Not applicable.
